# The Influence of Body Mass Composition on the Postural Characterization of School-Age Children and Adolescents

**DOI:** 10.1155/2018/9459014

**Published:** 2018-10-14

**Authors:** Wojciech Rusek, Joanna Baran, Justyna Leszczak, Marzena Adamczyk, Aneta Weres, Rafał Baran, Grzegorz Inglot, Teresa Pop

**Affiliations:** ^1^University of Rzeszow, Faculty of Medicine, Institute of Physiotherapy, Rzeszów, Poland; ^2^Rehabilitation Center REHAMED-CENTER, Tajęcina, Poland; ^3^RehaKlinika, Poland; ^4^Clinical Provincial Hospital No. 2, Rzeszów, Poland

## Abstract

**Introduction:**

In recent years a significant increase in the percentage of children with postural problems has been observed. It is necessary to focus on preventing the phenomenon and on analysis of existing postural defects.

**Aim:**

The aim of this work was to assess the potential relationship between body mass composition and body posture in school-age children.

**Material and Methods:**

464 school-age children ranging from 6 to 16 years (234 boys and 230 girls) were qualified for the study. Body mass composition was assessed using the analyzer Tanita MC 780 MA. Assessment of body posture was performed using Zebris system. All the results were analyzed with statistical methods. The accepted level of significance was p<0.05.

**Results:**

Analyses of the disparities between the girls and the boys showed statistically significant differences in all of the body mass components and in obliquity of the pelvis/shoulder. The boys were characterized by higher contents of muscle tissue (*p*<0.001), bone tissue (*p*<0.001), fatless tissue (*p*<0.001), and body water (*p*<0.001) as well as a greater obliquity angle (*p*=0.018). The girls, on the other hand, were found with higher content of fatty tissue (*p*<0.001). The children with lower content of muscle tissue (p=0.030), fatless tissue (p=0.030), water (p=0.030), and higher content of fatty tissue measured in kg (p=0.027) were characterized by greater pelvic obliquity.

**Conclusion:**

The current study shows evidence that sex, as a strongly differentiating factor, determines body mass composition and the occurrence of suboptimal postures only in the pelvic area. Body mass composition differentiates body posture of the study group. The content of fatty tissue influences the occurrence of suboptimal postures in the scapula and pelvic area in the frontal plane. The content of muscle tissue is associated with faulty postures in the scapula and pelvic area in the frontal plane.

## 1. Introduction

According to recent data published by WHO, overweight and obesity constitute a major public health problem in the 21st century. Worldwide prevalence of obesity has increased almost three times in the years 1975–2016. Childhood obesity is a global problem permanently occurring in numerous countries with low or medium income rates, especially in urban environments. The rate of incidence is increasing drastically. Globally, in 2016 it was estimated there were 41 million obese children below 5 years of age and over 340 million children aged 5-19 with excessive weight. Prevalence of overweight and obesity among children and teenagers aged 5-19 has increased from only 4% in 1975 to over 18% in 2016. This increase occurred both among boys (19%) and girls (18%). Slightly more than 1% of children and teenagers aged 5-19 years were obese in 1975, whereas in 2016, the problem was identified in 6% of girls and 8% of boys (in total over 124 million children and teenagers). Overweight and obesity cause a higher number of deaths worldwide compared to underweight. If the current trends continue by 2025 the number of overweight or obese infants and small children worldwide will increase to 70 million [1].

The increasing prevalence of excessive weight in school-age children may be one of the causes contributing to the growing problem of suboptimal postures in children. This justifies the need to examine school-age children, in order to verify whether there is a relationship between these two factors.

Suboptimal postures observed in children aged 3-18 years constitute one of the most common health problems worldwide. Aetiology is not homogeneous and additional negative factors include the fast pace of life in the contemporary world. There are numerous definitions of body posture and methods of its assessment. Body posture can be described by the relationship between the gravity line and body segments. Depending on the author of the relevant recommendations, assessment of body posture should include not only the vertical trunk position, but also the position of the shoulders, legs and feet, or the shape of the abdominal wall [2, 3].

Age is one of the main factors differentiating body posture. A child's body posture differs from an adult person's body posture. What is more, differences in body postures presented by children can vary relative to age [4]. Postural changes occur with high frequency in school-age children. Some of these reflect normal development of posture and are corrected as the child continues to grow. On the other hand, some of the changes are linked with asymmetries that can be caused by daily requirements of the body and can have a negative impact on the quality of life in childhood and adulthood [5, 6]. It is believed that particular threats to the body posture of a child occur during the periods of the fastest growth that is at the age of 6-7 years and in the period of puberty [7].

Varied degrees of suboptimal postures in Polish children are observed, depending on the region. Usually, these are associated with faulty habitual postures, to a large extent contributing to the increase in the population of individuals with suboptimal postures [8]. Wilmańska and others examined 285 children, Grade 1-3 pupils. They found suboptimal postures in 30.53% of the girls and 37.54% of the boys in the relevant population. An asymmetric position of the scapula was the most common body defect, identified in 37.77% of the girls and 31.33% of the boys. The greatest deviation from the norm was observed in the group of 7 years old [9].

Review of Polish data retrieved from the Healthcare Information Systems Centre (CSIOZ), based on Form MZ-11, ‘Report on operations and personnel working in basic outpatient healthcare', showed that spine deformations were identified in 4.5% of the population of children and teenagers (0-18 year) in 2005, and in 4.8% of the population in 2006. In 2007 those diagnosed with spine deformations accounted for 5.19% of the population. However, data for 2015, based on visits of children and teenagers consulting general practitioners (GP), showed that deformations of the spine were diagnosed in 5.4% of the children and teenagers aged 0-18. On the other hand, spine deformations most commonly occurred in children aged 10-14 [10]. For this reason, the authors' assessment of the relationship between the composition of body weight and the attitude in school-age children is a very important topic in the era of civilization diseases that affect a younger society.

## 2. Paper's Purpose

The primary aim of this study was to assess the relationship between sex and body composition and the attitude of the subjects.

The secondary aim of this study was to assess a potential relationship between body mass composition and body posture in school-age children.

## 3. Material and Method

The study was approved by the Bioethical Commission at the University of Rzeszów (approval no. 28/08/2016). The participants were informed about the course of the study. The examinations were conducted after written consent was received from school principals, parents of the children participating in the project and the children themselves.

### 3.1. Participants

464 school-age children, from 6 to 16, (234 boys and 230 girls), were qualified for the study, designed to be conducted in schools in Trzebownisko rural region. The schools participating were randomly selected. There are 9 primary schools and 7 secondary schools in the Trzebownisko commune. A total of 5 primary and 3 secondary schools participated in the study. Random selection was used. With the names of all 16 schools, 8 were selected to join the study.

The examinations were conducted in nurses' offices in the selected educational centres. In order to ensure reliability of the measurement, all the exams were performed by the same members of a qualified team, during morning hours. The subjects were asked not to eat or drink anything for six hours before the exam.

The following inclusion criteria were used: age from 6-18 years, place of residence in Trzebownisko, consent of parents and pupils for the examination.

Exclusion criteria were metal implants, electronic implants, menstruation, epilepsy, neurological and orthopaedic dysfunctions making it impossible to adopt a vertical position without aid, and failure to refrain from eating/drinking in the morning.

### 3.2. Anthropometric Measurements and Bioelectrical Impedance

Body height is measured with an accuracy of 0.1 cm using a portable stadiometer PORTSTAND 210. Body mass was measured with an accuracy of 0.1 kg. The measurements were conducted in standard conditions. For the examination, each participant, barefoot and dressed in underwear, was asked to assume upright extended position.

Body Mass Index (BMI in kg/m^2^) was calculated by dividing the body mass (kg) of each person by the squared height of his/her body (m^2^). The BMI value was analyzed in relation to the sex and age. The growth chart applied for the sex and age had been prepared within the frames of two Polish projects OLA and OLAF [11]. Based on the achieved percentile ranking the BMI status was classified in the following four categories of obesity (BMI ≥ 95. percentile), overweight (BMI ≥ 85. percentile and <95. percentile), normal body mass (BMI <85. percentile and ≥ 5. percentile), and underweight (BMI <5. percentile) [12].

Body mass composition was assessed using the analyzer Tanita MC 780 MA, whose operation is based on the phenomenon of bioelectrical impedance (BIA). The analyzer is approved for clinical use. It also has the certificate 93/42 EEC (EU norm for medical devices). Assessment of body mass composition took into account content of fatty tissue (FAT in % ang kg), fatless tissue (FFM in kg), muscle tissue (PMM in kg), bone tissue (BM in kg), and body water (TBW in kg).

### 3.3. Body Posture

**Figure 1:**
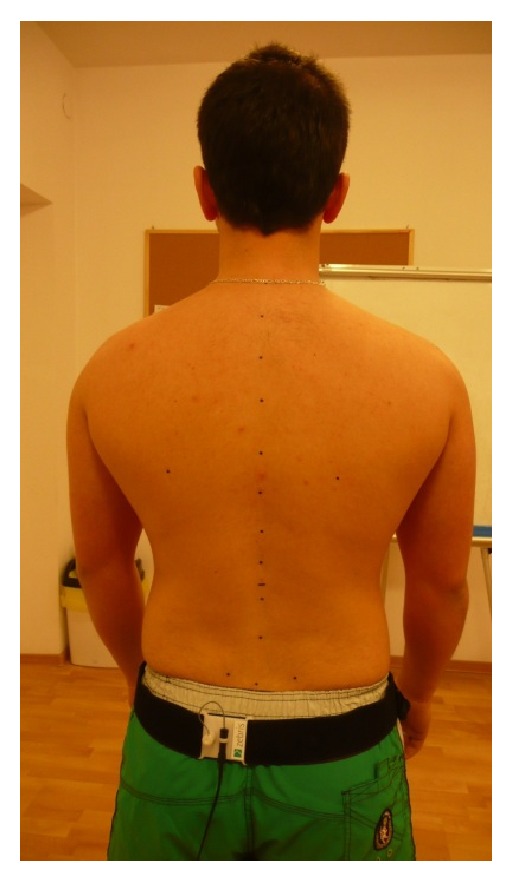
Subject's position during the examination.

The examination was conducted with the use of Zebris system, which consists of a measurement unit, system of microtransmitters and an ultrasound pointer, the latter applied to scan the topographic points from the skeleton. Topographical skeletal elements were marked using nonpermanent pen. The study was performed three times and the average result was analyzed. Before commencing the examination of the posture, the following skeletal topographic elements were marked: acromion right and left, posterior iliac spine right and left, anterior iliac spine right and left, iliac crest right and left, the point where the thoracic spine passes into the lumbar spine, Th 12/L1, inferior angle of the scapula right and left, and spinous process of the spine (Figure 1). The subject was in a free standing position, turned back to the measurement unit, without shoes and did not have with him/her any devices disturbing the transfer of data (e.g., smartwatch, mobile phone). The distance between the subject and the measuring device was 80 cm. A belt with a microtransmitter was placed on the shoulder girdle. The surface, i.e., the standing area, was calibrated for the subject; the measurement involved registration, by the ultrasound pointer, of the skeletal topographic points marked earlier. The exam was performed three times in every patient and the scan of the spine line was carried out nine times (three times in each study). The achieved result was the average of the measurements. The result of the study was saved in the computer program with the final report from the numerical data and graphs of the scanned points [13, 14].

The assessment of the posture took into an account

(i) in the sagittal plane, the distance of the right scapula (SDR in mm), the distance of the left scapula (SDL in mm), the difference in the distance of the scapula (SDD in mm), and pelvic torsion (PT in degree);

(ii) in the frontal plane, the difference in the height on the right side (PHDR in mm), the difference in the height on the left side (PHDL in mm), pelvic obliquity (PO in degree), and pelvic/shoulder obliquity (P/SO in degree).

### 3.4. Data Analysis

In order to assess whether there was a relationship between the parameters of body mass composition and body posture, Spearman's rank correlation test was used. The meaning of the intergroup differences in the selected parameters was examined using Kruskal-Wallis and Mann-Whitney tests. The accepted level of significance was* p*<0.05. All calculations and statistical analyses were performed with the use of STATISTICA ver. 10.0 (StatSoft, Poland).

## 4. Results

Detailed characteristics of the study group are presented in Tables 1 and 2. The mean age of the subjects was 11.52 years (SD=2.99). The average body height was 152.48cm (SD=17.77) and body mass 45.39kg (SD=16.81). Body mass index on average amounted to 18.8kg/m2 (SD=3.75) (Table 1).

Analyses of the disparities between the girls and the boys showed statistically significant differences in all of the body mass components and in obliquity of the pelvis/shoulder. The boys were characterized by higher contents of muscle tissue (*p*<0.001), bone tissue (*p*<0.001), fatless tissue (*p*<0.001), and body water (*p*<0.001) as well as greater obliquity angle (*p*=0.018). The girls, on the other hand, were found with higher content of fatty tissue (*p*<0.001) (Table 2).

Other analyses concerned the correlation between the parameters of the body mass composition and posture. A number of statistically significant effects were indicated. The children with the lower contents of muscle tissue (*p*=0.030), fatless tissue (*p=*0.030), and water (*p=*0.030) presented greater pelvic obliquity. They also were found with higher content of fatty tissue measured in kg (*p*=0.027).

Similarly, higher percentage of the fatty tissue content correlated with greater asymmetry in the scapula (*p=*0.025) and a higher position of the left shoulder (*p=*0.013). However, a reverse relation was observed between the content of fatty tissue (expressed in kg) and a higher position of the pelvis on the right side (*p*=0.015).

The analysis has also indicated, that the children with higher content of fatless tissue, muscle tissue and body water were characterized by pelvic asymmetry. This relation was found both for the right and the left side (accordingly* p*=0.004 for all the parameters and* p=*0.018 for all the parameters).

The final analysis showed that the children with higher contents of fatless tissue, muscle tissue and body water (*p=*0.016 for all the parameters) presented higher positioning of the right shoulder compared to the left one (Table 3).

## 5. Discussion

The current study shows evidence that sex, as a strongly differentiating factor, determines body mass composition and occurrence of suboptimal postures only in the pelvic area. Analysis of the correlations between postural parameters and body composition allowed to confirm the conclusions. The research allowed to assess the composition of body weight and body posture of the subjects. It was noticed that the content of adipose tissue influences the occurrence of postural defects in the region of the shoulder and pelvis in the frontal plane. On the other hand, the content of muscle tissue affects the occurrence of postural defects in the shoulders and pelvis in the frontal plane. In the available literature, the study of body posture and composition is rare, therefore continuing research in this area is extremely important.

The occurrence of overweight and obesity at school-age has become a great problem in the 21st century. Despite the fact that epidemiological data concerning the occurrence of suboptimal postures in school-age children represent varied levels, it is worrying that the percentage of children with excessive weight has been growing in recent years [15, 16]. In the present study, overweight and obesity was found in 20.5% of the boys and 17.9% of the girls. These rates do not correspond to the alarming tendencies of weight gain in the world. As many as 73% of the subjects had a BMI within the normal range. This may indicate that children from rural areas have better BMI parameters.

There are numerous methods of assessing body posture. However, both in Poland and internationally, researchers are constantly looking for more precise, reliable methods to assess suboptimal postures. Optimally, such methods would enable fast and at the same time accurate assessment. That is why in the present study we chose to examine body posture in the children in Trzebownisko using the Zebris system, a tool which can perform such assessment quickly, accurately, and reliably [13].

School environment, in which a child on average spends 6-8 hours per day, has a profound influence on his/her psychological and physical development. In addition to the hours spent sitting at the desk during classes, children spend more and more time in front of TV and computers, which may contribute to the growing occurrence of suboptimal postures and excessive weight [17].

In the available literature there are many studies discussing various factors which impact body posture in children (e.g., premature birth [18], weight of school backpacks [19], coexisting diseases [20, 21], overweight and obesity [22] and others). However, there is a lack of unambiguous evidence reflecting the relation between children's body posture and body mass composition. Additionally, most studies focusing on body posture assessment use subjective methods or the Moire method. There are few studies using the Zebris system, which is particularly recommended to be applied in assessing children because of its objectivity and short duration of the measurement.

The current study has allowed to check the frequency of suboptimal postures in the children attending schools in Trzebownisko, and to examine the effect of body mass composition in the occurrence of suboptimal postures.

Aleixo et al. evaluated changes in body posture of obese and overweight children aged 6-12 years. They wanted to determine if there are any correlations between, e.g., overloading of lower limbs or spine and suboptimal postures. They saw that there were differences in static and dynamic balance and praxis. They concluded that overweight and obesity impact body posture and balance in both aspects [23].

Maciałczyk-Paprocka et al. reported that excessive body mass was connected with suboptimal postures in children at the age of 7-12. The most common deviations from normal posture in the obese children and teenagers included genu valgum and flat foot [3].

Wyszyńska et al. established that body mass components can influence a variety of postural parameters. For instance fat content determines the variability in the following parameters: obliquity of the thoracic-lumbar segment, angle of the shoulder line and the position of the scapula. Muscle tissue content explains the variability in the parameter specifying the mutual scapula position [4]. However, the current study showed that higher content of fatty tissue was associated with greater asymmetry in the scapular area.

Rusek and colleagues studied a group of young adolescents in order to determine the factors influencing changes in body posture. In their study they used the Zebris system. They reported statistically significant relations of body posture with sex, playing musical instruments, doing sports and presence of vision defects [13]. Studies show the dependence of disorders in body posture in people who for many years have been playing musical instruments that require asymmetric muscle work. People playing the guitar spend 1 to 2 hours a day on the training a few times a week [24, 25]. Also, practicing sports with an asymmetrical positioning of the player's body causes deformation of the spine in the frontal plane and changes in the head's act. If left untreated, they may cause changes in the shoulder, chest and pelvis settings and, in the convention, change into permanent ones [26]. In own studies, this type of dependence was not analyzed, they will be the subject of further research.

Kapo et al. presented results of a study involving 52 children aged 5-14, which showed evidence that there is a negative trend leading to an increase in body mass index from an early age. These authors noticed the children presented a tendency towards increasing deformations of the shoulder line and a tendency to develop a mild form of kyphotic posture. The other type of deformations, visible during the exams, was connected with a trend towards increased pelvic rotation, which leads to lordotic posture in all three age groups (5-8 years, 9-11 years, 12-14 years). Additionally, the authors analyzed the correlation between the measured and the registered variables and found that there was no significant correlation between the subjects' BMI and the deviations in their postural status. However, there is a close correlation between some specific deformations which suggests that one body deformation to a large extend determines development of another deformation; this commonly happens in connected segments where a deviation in the normal postural status is caused by the dysfunction [27].

Those results are consistent with the current findings which also show a close correlation between body mass composition and specific body posture disorders.

The aim of a study Schwanke et al. was to investigate changes in body posture and muscle strength in obese and overweight children and adolescents as a result of exercise. They evaluated 46 children and saw that a specific exercise program, with global postural training, contributed to improved posture and greater strength of abdominal muscle [28].

Results by other authors shows that there was an inverse association between bone physical properties and a flat posture. Bone and posture were more strongly positively linked in a rounded posture. This suggest that both bone properties and posture mature in a shared and interrelated mechanical environment, probably modulated by pattern-specific anthropometrics and body composition [29]. Adult sagittal posture is established during childhood and adolescence. A flattened or hypercurved spine is associated with poorer musculoskeletal health in adulthood. Although anthropometry from birth onwards is expected to be a key influence on sagittal posture design, this has never been assessed during childhood. Araújo et al. findings support a prospective sculpting role of body size and therefore of load on musculoskeletal spinopelvic structures, with stronger associations as children get older [30]. Although the Zebris system is a very effective tool, it also has its limitations. The first is the cost of the related equipment. Finally, because of the considerable duration of the exam, sometimes patients can move and falsify the test result. That is why it is so important to properly prepare both the subject and the researcher.

The achieved results confirm a need to conduct a controlled study designed to detect suboptimal postures in children. Despite the numerous health-related educational programs, the problem of suboptimal postures is not decreasing but it continues to grow. Normal development of young people should be a matter of concern for parents and teachers as well as local authorities; that is why cooperation in this area, at all levels, is crucial.

## 6. Conclusions

The current study shows evidence that sex, as a strongly differentiating factor, determines body mass composition and occurrence of suboptimal postures only in the pelvic area.

Body mass composition differentiates body posture of the study group. The content of fatty tissue influences the occurrence of suboptimal postures in the scapula and pelvic area in the frontal plane. The content of muscle tissue is associated with faulty postures in the scapula and pelvic area in the frontal plane.

## Figures and Tables

**Table 1:** Characteristics of the study group.

	**n**	**M**	**Me**	**SD**	**Q**_**1**_	**Q**_**3**_
**All**						

**Age [years]**	464	11.52	12.00	2.99	9.00	14.00

**Height [cm]**	464	152.48	155.50	17.77	137.00	167.00

**Weight [kg]**	464	45.39	44.75	16.81	30.40	56.45

**BMI [kg/m** ^**2**^ **]**	464	18.80	18.24	3.75	16.10	20.97

**Girls **						

**Age [years]**	230	11.55	12	2.96	9.00	14.00

**Height [cm]**	230	150.46	155	15.69	136.00	163.00

**Weight [kg]**	230	43.63	44.35	15.38	29.70	54.60

**BMI [kg/m** ^**2**^ **]**	230	18.64	18.00	3.87	15.90	20.90

**Boys **						

**Age [years]**	234	11.49	12	3.02	9.00	14.00

**Height [cm]**	234	154.46	156.50	19.43	138.00	172.00

**Weight [kg]**	234	47.13	45	17.97	31.80	60.00

**BMI [kg/m** ^**2**^ **]**	234	18.94	18.56	3.63	16.40	21.05

M: mean; Me: median; n: number of subjects; Q_1_: first quartile; Q_3_: third quartile SD: standard deviation.

**Table 2:** The difference in body posture parameters between the boys and the girls.

	**Girls**						**Boys**						***p***
	**n**	**M**	**Me**	**SD**	Q_1_	Q_3_	**n**	**M**	**Me**	**SD**	Q_1_	Q_3_	
**FAT [**%**]**	230	23.96	23.40	5.52	19.50	27.20	234	19.30	17.60	5.77	15.40	21.70	**<0.001**

**FAT [kg]**	230	11.07	9.55	6.24	6.20	14.40	234	9.44	8.20	5.85	5.30	11.20	**0.001**

**FFM [kg]**	230	32.56	34.00	9.76	22.90	39.80	234	37.62	36.35	13.43	25.90	49.30	**<0.001**

**TBW [kg]**	230	23.83	24.90	7.14	16.80	29.10	234	27.54	26.60	9.83	19.00	36.10	**<0.001**

**PMM [kg]**	230	30.88	32.25	9.28	21.70	37.80	234	35.65	34.45	12.82	24.50	46.80	**<0.001**

**BM [kg]**	230	1.68	1.75	0.48	1.20	2.00	234	1.97	1.90	0.61	1.40	2.50	**<0.001**

**Pelvic torsion [degree]**	230	3.72	2.90	3.36	1.40	4.80	234	3.59	2.85	3.50	1.30	4.80	0.561

**Pelvic obliquity [degree]**	230	2.04	1.60	1.83	0.70	2.80	234	2.18	1.95	1.71	0.80	3.00	0.110

**Pelvic/ shoulder obliquity [degree]**	230	2.38	1.90	1.85	1.00	3.40	234	3.55	2.50	10.86	1.20	4.10	**0.018**

**Scapula distance difference [mm]**	230	6.94	5.00	5.83	3.00	10.00	234	7.18	6.00	6.46	3.00	9.00	0.988

**Pelvic height difference right [mm]**	229	3.85	0.00	6.24	0.00	5.80	234	4.95	0.40	6.98	0.00	9.00	0.173

**Pelvic height difference left [mm]**	230	4.17	0.20	6.53	0.00	6.10	234	3.98	0.00	6.37	0.00	6.10	0.729

**Shoulder height difference right [mm]**	83	6.24	4.80	5.50	2.50	8.40	67	6.79	6.60	4.93	3.10	9.40	0.183

**Shoulder height difference left [mm]**	146	11.83	10.25	8.04	5.30	17.30	167	11.38	9.40	8.84	4.50	16.20	0.438

BM [kg]: bone mass; Fat [%]: body fat percentage; Fat [kg]: mass of body fat; FFM[kg]: free-fat mass; M: mean; Me: median; n: number of subjects; *p*: Mann–Whitney test probability value; PMM [kg]: muscle mass; Q1: first quartile; Q3: third quartile; SD: standard deviation; TBW [kg]: total body water mass.

**Table 3:** Correlation analysis for the parameters of body posture and body mass composition.

	**R**	**p**
**Pelvic torsion [degree] & Fat [**%**]**	0.01	0.879

**Pelvic torsion [degree] & Fat [kg]**	0.02	0.649

**Pelvic torsion [degree] & FFM [kg]**	-0.03	0.497

**Pelvic torsion[degree] & TBW [kg]**	-0.03	0.499

**Pelvic torsion [degree] & PMM [kg]**	-0.03	0.501

**Pelvic obliquity[degree] & Fat [**%**]**	0.06	0.193

**Pelvic obliquity [degree] & Fat [kg]**	0.10	**0.027**

**Pelvic obliquity[degree] & FFM [kg]**	-0.10	**0.030**

**Pelvic obliquity [degree] & TBW [kg]**	-0.10	**0.030**

**Pelvic obliquity[degree] & PMM [kg]**	-0.10	**0.030**

**Pelvic/shoulder obliquity [degree] & Fat [**%**]**	-0.05	0.247

**Pelvic/shoulder obliquity[degree] & Fat [kg]**	-0.03	0.522

**Pelvic/shoulder obliquity [degree] & FFM [kg]**	0.02	0.688

**Pelvic/shoulder obliquity [degree] & TBW [kg]**	0.02	0.687

**Pelvic/shoulder obliquity[degree] & PMM [kg]**	0.02	0.689

**Scapula distance difference [mm] & Fat [**%**]**	0.10	**0.025**

**Scapula distance difference [mm] & Fat [kg]**	0.07	0.133

**Scapula distance difference [mm] & FFM [kg]**	-0.01	0.890

**Scapula distance difference [mm] & TBW [kg]**	-0.01	0.892

**Scapula distance difference [mm] & PMM [kg]**	-0.01	0.893

**Pelvic height difference [mm] right & Fat [**%**]**	-0.03	0.461

**Pelvic height difference [mm] right & Fat [kg]**	-0.11	**0.015**

**Pelvic height difference [mm] right & FFM [kg]**	0.13	**0.004**

**Pelvic height difference [mm] right & TBW [kg]**	0.13	**0.004**

**Pelvic height difference [mm] right & PMM [kg]**	0.13	**0.004**

**Pelvic height difference [mm] left & Fat [**%**]**	-0.03	0.584

**Pelvic height difference [mm] left & Fat [kg]**	-0.09	0.057

**Pelvic height difference [mm] left & FFM [kg]**	0.11	**0.018**

**Pelvic height difference [mm] left & TBW [kg]**	0.11	**0.018**

**Pelvic height difference [mm] left & PMM [kg]**	0.11	**0.018**

**Shoulder height difference [mm] right & Fat [**%**]**	-0.02	0.837

**Shoulder height difference [mm] right & Fat [kg]**	0.15	0.073

**Shoulder height difference [mm] right & FFM [kg]**	0.20	**0.016**

**Shoulder height difference [mm] right & TBW [kg]**	0.20	**0.016**

**Shoulder height difference [mm] right & PMM [kg]**	0.20	**0.016**

**Shoulder height difference [mm] left & Fat [**%**]**	0.14	**0.013**

**Shoulder height difference [mm] left & Fat [kg]**	0.10	0.091

**Shoulder height difference [mm] left & FFM [kg]**	0.03	0.540

**Shoulder height difference [mm] left & TBW [kg]**	0.03	0.538

**Shoulder height difference [mm] left & PMM [kg]**	0.03	0.541

BM [kg]: bone mass; Fat [%]: body fat percentage; Fat [kg]: mass of body fat; FFM [kg]: free-fat mass; *p*: Mann–Whitney test probability value; PMM [kg] muscle mass; R: Spearman's rank correlation coefficient; TBW [kg]: total body water mass.

## Data Availability

The results from our study used to support the findings of this study are included within this article. The personal data used to support the findings of this study are restricted by the Bioethical Committee in University of Rzeszow in order to protect patient privacy. Data are available from corresponding author for researchers who meet the criteria for access to confidential data.
